# Physiological and Dietary Determinants of Iron Status in Spanish Vegetarians

**DOI:** 10.3390/nu11081734

**Published:** 2019-07-26

**Authors:** Angélica Gallego-Narbón, Belén Zapatera, M. Pilar Vaquero

**Affiliations:** Department of Metabolism and Nutrition, Institute of Food Science, Technology and Nutrition (ICTAN-CSIC), José Antonio Novais, 10, 28040 Madrid, Spain

**Keywords:** iron deficiency, iron status, body iron, ferritin, menstruation, vegetarian, vegan, supplementation

## Abstract

Vegetarian diets may compromise iron status, as they provide non-haem iron which has low bioavailability. Spanish lacto-ovo vegetarians (*n* = 49) and vegans (*n* = 55) were recruited and haematological and biochemical iron parameters were analysed. Food and supplements consumption, body composition, physical activity, menstrual blood losses and hormonal contraceptive use were assessed. Four groups were studied: Iron deficiency anaemia (IDA), iron depletion (ferritin <15 ng/mL), iron deficiency (ferritin ≥15 to ≤30 ng/mL), and iron sufficiency (ferritin >30 ng/mL). IDA was uncommon (*n* = 5, 4.8%), 27.9% of participants were iron-depleted, and 30.8% were iron-deficient. Serum ferritin was lower in women than men (*p* < 0.001) and IDA and iron depleted individuals were all women. There were no differences attributed to diet type, time being vegetarian or physical activity. The menstrual period length was negatively associated with transferrin saturation (*ρ* = −0.364, *p* = 0.001) and hormonal contraceptive use (*ρ* = −0.276, *p* = 0.014). Iron supplements were consumed most frequently by IDA and iron-deficient subjects (*p* = 0.031). Conclusions: Iron status did not vary between lacto-ovo vegetarians and vegans and there was not an influence of the time following a vegetarian diet. Although men were iron-sufficient, iron deficiency was frequent in women, who should apply strategies to increase iron bioavailability, especially if they experience intense menstrual blood losses.

## 1. Introduction

Appropriately planned vegetarian diets provide health benefits, but they are also associated with a higher risk of iron deficiency than omnivorous diets [1]. The maintenance of adequate levels of this micronutrient is essential for oxygen transport, energy storage, and protein synthesis [2]. Iron deficiency may lead to iron deficiency anaemia (IDA), activate bone resorption, affect the immune system, and limit physical activity [2,3]. On the opposite side, excessive iron deposits in the organism have been linked with cardiometabolic alterations, including obesity and metabolic syndrome [4].

The most specific measure of iron stores is serum ferritin, although additional tests are recommended. Transferrin and soluble serum transferrin receptor (sTfR), considered early indicators of functional iron deficiency, are related to iron transport and their concentrations are increased with iron depletion [5,6,7]. Prolonged insufficient iron supply to tissues eventually leads to IDA, characterised by low haemoglobin, ferritin, transferrin saturation (TSAT), mean corpuscular haemoglobin (MCH) and mean corpuscular volume (MCV) [3,5]. When body iron decreases, iron absorption increases, as the synthesis of hepcidin, a downregulator of iron absorption, is stimulated [2,6].

In this regard, vegetarian diets (including the vegan type) do not necessarily imply a low iron intake, as they include iron-rich products, such as legumes, integral cereals, nuts, and green-leafy vegetables [8]. However, plants contain exclusively non-haem iron, with low bioavailability and whose absorption is dependent on the balance between iron absorption enhancers and inhibitors [6]. Among the enhancing factors, meat, absent in vegetarian diets, and vitamin C, highly consumed in plant-based diets, are the most important [5,9]. Nevertheless, vegetarian diets are also rich in absorption inhibitors, including phytates (present in whole grains, integral cereals and legumes), polyphenols (present in coffee, tea and wine) and calcium [2,10]. Regarding iron status, previous research shows that vegetarians have lower ferritin than non-vegetarians, but haemoglobin is usually similar in both groups [1]. The absence of differences in IDA prevalence suggests an enhanced iron absorption in vegetarians due to up-regulation.

Menstrual blood loss constitutes an additional determinant of iron status in women, as its association with IDA has been demonstrated [11], but this factor is usually not taken into account in research studies. In relation to this, hormonal contraceptive use reduces blood loss and should also be considered in studies on iron status [12]. While non-menstruating women and men lose approximately 1 mg of iron daily, menstruating women lose 10 to 42 extra mg per menstrual cycle [6]. This loss leads to lower iron status in women of reproductive age than in men independently of their diet [13]. Menstruation could be of special interest in vegetarian women, as previous reports suggest that they present menstrual alterations [14].

The nutritional status of Spanish vegetarians has only begun to be addressed and their iron status has never been studied. The estimates indicate that 1.5% of Spaniards follow a vegetarian diet [15] and due to their scarce representation, the health status of this group has remained unexplored. The recent data on food consumption of Spanish adults showed the number of non-fish consumers and non-meat consumers, but not the number of people who did not consume any of them [16]. This suggests that the proportion of vegetarians and their needs could have been underestimated.

This research evaluated the iron status of Spanish vegetarians using haematological and biochemical markers, and studied the relations of iron stores with physical activity, menstruation, iron supplementation, and dietary patterns.

## 2. Materials and Methods

### 2.1. Participants

Healthy adult (age ≥18 years) lacto-ovo vegetarians and vegans were recruited in the Madrid area (Spain) through online advertisements. The exclusion criteria were: Consumption of meat or fish, eating disorders, pregnancy, lactation, menopause, and diagnosed diseases (digestive, renal, haematological, endocrine or oncological). In addition, volunteers were not included if they had donated blood during the three months prior to the study. A total of 207 subjects were interested in the study, of which 44 decided not to participate and 58 did not meet the inclusion criteria. Therefore, 105 volunteers were selected, of which 104 underwent the biochemical analyses and provided complete data.

The study followed the Declaration of Helsinki guidelines and procedures requiring human subjects were approved by the ethics committees of Hospital Puerta de Hierro (Majadahonda, Spain) and Spanish National Research Council (CSIC). A written informed consent was obtained from all the participants.

### 2.2. Procedure and Determinations

The participants attended the institute after a fasting period of 12 h. The height and abdominal and hip perimeters were measured. The body weight and body composition were obtained through the body composition monitor Tanita BC-601 (Tanita Ltd., Amsterdam, The Netherlands), body mass index (BMI) and waist-hip ratio (WHR) were calculated. The blood samples were collected and leucocytes, erythrocyte count (RBC), haemoglobin, haematocrit, MCV, MCH, mean corpuscular haemoglobin concentration (MCHC), erythrocyte distribution width (RDW), platelets, and mean platelet volume (MPV) were measured in the flow cytometer ADVIA 2120i (Siemens, Erlangen, Germany).

The serum samples were separated by centrifugation at 1000 g for 15 min in a Jouan CR-312 centrifuge (Jouan Ltd, Ilkeston, UK). The serum iron and transferrin were analysed in an ADVIA Chemistry XPT clinical chemistry system and ferritin in an ADVIA Centaur XP analyser (Siemens Healthineers, USA). sTfR was measured using an ELISA kit (DRG Instruments, Marburg, Germany). The total iron binding capacity (TIBC) was calculated as 25.1 × serum transferrin (g/L), and TSAT as serum iron (µmol/L)/TIBC × 100. sTfR/ferritin ratio was calculated and total body iron (TBI) was obtained as: −[log (sTfR/ferritin) − 2.8229]/0.1207 [17].

### 2.3. Diet and Health Questionnaires

Dietary habits were studied through an optimized version of a previously published 160-item food frequency questionnaire for self-defined vegans in the United States [18], that also permitted the classification of volunteers as lacto-ovo vegetarians or vegans. The period of vegetarianism (<1 year, 1–3 years, 4–10 years, > 10 years), food frequency consumption (never or rarely, 2–4 times/month, 2–3 times/week, 4–6 times/week, once a day, ≥2 times/day), and iron supplementation (never, 1–12 times/year, 2–5 times/month, 2–6 times/week, daily consumed) were evaluated. The foods with a mean consumption ≥2–4 times/month that were iron sources (≥2.1 mg/100 g), vitamin C sources (≥12 mg/100 g) or contained iron absorption inhibitors (phytates or polyphenols), were selected for statistical analysis. Iron and vitamin C food sources were obtained from the Spanish Food Composition Database (BEDCA) and the Commission Directive 2008/100/EC [19,20]. Physical activity was estimated by the short form of the international physical activity questionnaire (IPAQ). Menstruation and hormonal contraceptive use were assessed by a gynaecological questionnaire [21].

### 2.4. Statistical Analysis

Normality was studied through Kolmogorov-Smirnov tests and variables with non-normal distributions were log-transformed. The differences between genders and diet types (lacto-ovo vegetarian or vegan) in continuous variables were analysed by generalized linear models (GLM). The volunteers were classified in four groups: IDA (haemoglobin <13 g/dL for men or <12 g/dL for women), depletion (ferritin <15 ng/mL), iron deficiency (ferritin ≥15 to ≤30 ng/mL), and iron sufficiency (ferritin >30 ng/mL) [22,23]. The differences among groups were analysed by Kruskal-Wallis or Pearson’s χ^2^ tests corrected by 10,000 Monte-Carlo simulations with a confidence interval of 99%. The association among gynaecological variables and iron-status markers were studied by Spearman´s correlations. The statistical analyses were performed in SPSS 24 (SPSS Inc., Chicago, IL, USA) with significance level of *p* < 0.05.

## 3. Results

### 3.1. Characteristics of the Participants

The participants were 30.3 ± 7.7 (mean ± SD) years old lacto-ovo vegetarians (49 subjects, 47.1%) or vegans (55 subjects, 52.9%), and the majority were women (81 subjects, 77.9%; Table 1). The volunteers were normoweight, with moderate to high physical activity and low consumption of iron supplements. BMI (*p* = 0.030), WHR, body muscle weight, bone mass, water percentage, and abdominal muscle (*p* < 0.001) were higher in men, while body fat (*p* < 0.001) and abdominal fat (*p* = 0.006) were higher in women. The differences in physical activity, iron supplementation and body composition between diet groups were not detected.

The levels of the studied markers are shown in Table 2. RBC, haemoglobin, haematocrit, TSAT, ferritin, TBI, MCHC, and serum iron were significantly higher in men, while RDW, transferrin, and sTfR/ferritin ratio were significantly higher in women. MCH was slightly higher in vegans (*p* = 0.041) but no other significant differences between lacto-ovo vegetarians and vegans were found.

### 3.2. Iron Status, Menstruation, and Consumption Patterns

Table 3 shows the differences in iron biomarkers, iron supplementation, body composition and vegetarian patterns of the four iron status groups. The proportion of iron sufficient individuals was low (*n* = 38, 36.5%), including most of the men (*n* = 20, *p* < 0.001). The proportion of lacto-ovo vegetarians, period of vegetarianism and physical activity was not significantly different among the groups. WHR, body muscle, bone mass and abdominal muscle were higher in iron sufficient individuals (*p* < 0.001). The menstruation parameters and hormonal contraception use did not vary among the groups (Table 4). Further, 25% of the women used hormonal contraceptives. The menstrual period length and heavy bleeding days were negatively associated (Spearman´s correlation) with hormonal contraceptive use (*ρ* = −0.276, *p* = 0.014; and *ρ* = −0.260, *p* = 0.021) and TSAT (*ρ* = −0.364, *p* = 0.001; and *ρ* = −0.285, *p* = 0.011). The frequency of consumption of peas (*p* = 0.013), zucchini (*p* = 0.005), and pasta (*p* = 0.030) were higher in individuals with lower iron stores (Table 5).

## 4. Discussion

This study examined the iron status of Spanish vegetarians for the first time. The results reveal that IDA prevalence was low, consistent with previous reports in vegetarians from developed countries [13,24,25]. In order to detect iron deficiency before the appearance of IDA, sTfR in addition to the classical iron markers was determined. This marker, in contrast to ferritin, is not affected by inflammation and is increased in the first stages of iron deficiency. The sTfR/ferritin and TBI indexes were also calculated, that have previously proved to be useful in iron deficiency diagnosis [26]. In this regard, sTfR/ferritin decreases as iron status increases in the studied subjects. The dietary characteristics, physical activity and menstruation patterns were also evaluated and allowed the identification of the main predictors of iron status in this population.

Vegetarian men results indicate iron sufficiency, as none of them were anaemic and the cases of low iron status were anecdotic (*n* = 3, 13%). In contrast, approximately 78% of women exhibited iron deficiency or depletion. These results are in line with other studies in lacto-ovo vegetarian and vegan women from different developed countries that obtained ferritin levels ranging from 14 to 26 ng/mL [24,25,27]. The present results show lower iron status than observed in a recent study in German vegetarians [28]. The higher iron status may be expected in vegans as dairy products reduce iron bioavailability [2,29], but similar rates of iron deficiency and iron depletion in both diet groups were observed, which can be explained by the low consumption of milk of the lacto-ovo vegetarian participants [30]. Moreover, the dietary patterns were not clearly different in the four iron status groups. The highest consumption of several iron and vitamin C rich foods, such as peas, was observed in the IDA group. However, this group also presented a high consumption of iron absorption inhibitors such as pasta, which included integral pasta and was generally served with spices (oregano, pepper, basil, etc.) which are considered inhibitors [2]. These results suggest that iron bioavailability is dependent on the consumption of absorption enhancers and inhibitors and their combination in the diet [31].

Therefore, the type of vegetarian diet, lacto-ovo vegetarian or vegan, appears to have a minor effect. The iron status of women can be mainly attributed to menstruation, in line with previous findings [11,21]. The menstruation length was negatively associated with TSAT, and anaemic individuals tended to have longer menstruations than the rest of women. These women also reported not being hormonal contraceptive users, which is consistent with the negative association between hormonal contraceptive use and menstruation length hereby observed and with previous studies demonstrating that contraceptives can reduce menstrual blood loss [21]. Interestingly, iron supplementation was more frequent in groups with low iron status than in the iron-sufficient group, suggesting an awareness of IDA risk in these vegetarians. In order to prevent iron deficiency, foods containing iron absorption inhibitors (such as bran, coffee and tea) should be ingested separately from meals providing non-haem iron, and foods containing iron enhancers should be consumed with the main iron containing meal. In addition, women with high menstrual blood losses, at risk of iron deficiency, should check their iron stores and haematological parameters regularly.

In this survey, sedentary subjects represented less than 10% of the participants. Low physical activity is expected in anaemic subjects, which is consistent with the obtained results [23]. In addition, the performance of intense exercise is associated with low ferritin, especially in women with intense menstrual losses [32]. The results on physical activity and body composition should be interpreted considering gender differences, as men naturally exhibit higher muscle and bone mass than women. Nevertheless, although physical activity was not higher in men, the highest proportion of active subjects was observed in the iron-sufficient group including most of the men, and the lowest in the IDA group.

Finally, an association between the period of vegetarianism and iron status was not detected, with iron deficiency present in short-term and long-term vegetarians. The lower iron status that long-term vegetarians could potentially exhibit might be masked by more significant factors, including menstrual blood loss or adaptation to a low iron status by an increased absorption.

This study has several limitations. The sample is not representative of the Spanish vegetarian population, since the number of vegetarians in this country is unknown, and a control group of omnivores has not been included for comparison. In addition, the dietary assessment was performed through a food frequency questionnaire and the collected dietary data were not quantitative. However, its main strength is that all iron status biomarkers were analysed, and dietary, physiological and behavioural factors were also considered, allowing a comprehensive study of iron status.

## 5. Conclusions

Insufficient iron status was detected in vegetarian women, being menstruation and hormonal contraceptive use the main predictors, while men were generally iron sufficient. The influence of the type of vegetarian diet was negligible, probably due to the balance between iron absorption enhancers and inhibitors, and iron supplementation practice was noticed in anaemic and iron deficient women. Although the prevalence of IDA in this population is low, menstruating women should combine foods properly to prevent iron deficiency. Our results suggest that nutritional supplements should not be widely recommended to vegetarians, although women with intense menstrual blood losses should apply dietary strategies to improve iron bioavailability, i.e., consume iron absorption enhancers with the main meals, while iron inhibitors separated from the main meals, and regularly check their iron status in order to know if they need an iron supplement.

## Figures and Tables

**Table 1:** The characteristics of the participants.

	Women			Men				
	LO-V (*n* = 38)	Vegans (*n* = 43)	All (*n* = 81)	LO-V (*n* = 11)	Vegans (*n* = 12)	All (*n* = 23)	*P*_G_ *	*P*_D_ *
Age (year)	30.6 (8.1)	28.7 (6.9)	29.6 (7.5)	33.5 (9.0)	32.3 (7.3)	32.8 (8.0)	0.081	0.521
	Iron supplementation						0.175	0.961
Never	30 (78.9)	35 (81.4)	65 (80.2)	11 (100.0)	11 (91.7)	22 (95.7)		
1–12 times/year	5 (13.2)	6 (14.0)	11 (13.6)	0 (0.0)	0 (0.0)	0 (0.0)		
2–5 times/month	1 (2.6)	2 (4.7)	3 (3.7)	0 (0.0)	0 (0.0)	0 (0.0)		
1–6 times/week	0 (0.0)	0 (0.0)	0 (0.0)	0 (0.0)	0 (0.0)	0 (0.0)		
Daily	2 (5.3)	0 (0.0)	2 (2.5)	0 (0.0)	1 (8.3)	1 (4.3)		
	Physical activity level						0.767	0.913
Low	2 (5.3)	4 (9.3)	6 (7.4)	2 (18.2)	0 (0.0)	2 (8.7)		
Moderate	17 (44.7)	20 (46.5)	37 (45.7)	3 (27.3)	5 (41.7)	8 (34.8)		
High	19 (50.0)	19 (44.2)	38 (46.9)	6 (54.5)	7 (58.3)	13 (56.5)		
WHR	0.80 (0.0)	0.80 (0.1)	0.80 (0.1)	0.92 (0.1)	0.89 (0.1)	0.90 (0.1)	**<0.001**	0.684
BMI (kg/m^2^)	22.8 (3.8)	21.7 (2.5)	22.2 (3.2)	25.0 (5.0)	23.0 (2.5)	24.0 (3.9)	**0.030**	0.125
Body fat (%)	26.1 (6.8)	25.1 (6.1)	25.6 (6.4)	16.1 (7.8)	13.7 (5.0)	14.8 (6.5)	**<0.001**	0.342
Body muscle (kg)	41.5 (3.5)	40.7 (2.9)	41.1 (3.2)	60.4 (8.0)	58.7 (6.7)	59.5 (7.2)	**<0.001**	0.450
Bone mass (kg)	2.2 (0.2)	2.2 (0.2)	2.2 (0.2)	3.2 (0.4)	3.1 (0.3)	3.1 (0.3)	**<0.001**	0.348
Body water (%)	54.4 (4.7)	55.2 (4.5)	54.9 (4.6)	60.9 (7.0)	61.8 (3.9)	61.4 (5.5)	**<0.001**	0.311
Abdominal fat (%)	21.7 (8.3)	20.4 (7.6)	21.0 (7.9)	17.1 (9.5)	13.9 (6.5)	15.5 (8.0)	**0.006**	0.217
Abdominal muscle (kg)	24.3 (3.1)	23.4 (1.7)	23.8 (2.5)	32.5 (4.5)	32.1 (4.0)	32.3 (4.1)	**<0.001**	0.386

The results expressed as the mean (SD) for continuous variables and *n* (%) for categorical variables. D, diet type; G, gender; LO-V, lacto-ovo vegetarians; * GLM or Pearson’s χ^2^ test. Significant differences are in bold.

**Table 2:** The haematological and biochemical markers according to gender and diet type.

	Women			Men				
	LO-V (*n* = 38)	Vegans (*n* = 43)	All (*n* = 81)	LO-V (*n* = 11)	Vegans (*n* = 12)	All (*n* = 23)	*P*_G_ *	*P*_D_ *
Leucocytes (10^3^/µL)	6.1 (1.6)	5.9 (1.7)	6.0 (1.6)	5.8 (1.8)	5.7 (1.4)	5.8 (1.6)	0.411	0.697
RBC (10^6^/µL)	4.5 (0.3)	4.4 (0.3)	4.5 (0.3)	5.0 (0.5)	4.9 (0.3)	4.9 (0.4)	**<0.001**	0.163
Haemoglobin (g/dL)	13.5 (1.2)	13.5 (0.9)	13.5 (1.1)	15.2 (1.0)	15.7 (0.8)	15.4 (0.9)	**<0.001**	0.691
Haematocrit (%)	41.1 (3.2)	40.8 (2.9)	40.9 (3.1)	45.0 (2.7)	45.9 (2.6)	45.5 (2.6)	**<0.001**	0.952
MCV (fL)	90.6 (5.9)	93.1 (4.6)	91.9 (5.4)	91.1 (6.0)	93.4 (2.9)	92.3 (4.7)	0.928	0.431
MCH (pg)	29.7 (2.5)	30.8 (1.6)	30.3 (2.1)	30.7 (1.8)	31.9 (1.2)	31.3 (1.6)	0.079	**0.041**
MCHC (g/dL)	32.8 (1.3)	33.1 (0.9)	33.0 (1.1)	33.7 (0.8)	34.2 (1.1)	33.9 (1.0)	**0.002**	0.438
RDW (%)	13.8 (1.5)	13.2 (0.9)	13.5 (1.3)	12.9 (0.6)	12.7 (0.4)	12.8 (0.5)	**0.004**	0.180
Platelets (10^3^/µL)	252 (61)	241 (75)	246 (69)	241 (59)	188 (52)	213 (61)	0.080	0.078
MPV (fL)	10.0 (1.3)	10.2 (1.3)	10.1 (1.3)	9.7 (0.7)	10.1 (1.1)	9.9 (0.9)	0.488	0.431
Iron (µmol/L)	14.4 (8.2)	17.3 (8.0)	15.9 (8.2)	19.9 (6.3)	22.0 (8.1)	21.0 (7.2)	**0.011**	0.287
Transferrin (g/L)	3.1 (0.5)	3.3 (0.6)	3.2 (0.6)	2.7 (0.2)	2.6 (0.3)	2.6 (0.3)	**<0.001**	0.940
TSAT (%)	19.0 (12.2)	21.8 (10.6)	20.5 (11.4)	29.5 (8.3)	33.6 (10.4)	31.6 (9.4)	**<0.001**	0.240
Ferritin (ng/mL)	21.1 (14.3)	21.9 (16.1)	21.5 (15.2)	81.8 (68.8)	71.4 (19.1)	76.3 (48.6)	**<0.001**	0.617
sTfR (mg/L)	0.9 (0.6)	0.8 (0.3)	0.8 (0.3)	0.8 (0.3)	0.8 (0.3)	0.8 (0.4)	0.364	0.641
sTfR/ferritin	67.4 (79.0)	70.7 (91.7)	69.2 (85.5)	26.8 (32.9)	12.3 (5.6)	19.2 (23.7)	**<0.001**	0.452
TBI (mg/kg)	9.9 (3.5)	9.9 (3.4)	9.9 (3.4)	13.9 (4.3)	14.7 (1.8)	14.4 (3.2)	**<0.001**	0.452

The results expressed as the mean (SD). D, diet type; G, gender; LO-V, lacto-ovo vegetarians. * GLM. Significant differences are in bold.

**Table 3:** The vegetarian pattern, iron supplementation, physical activity, body composition, and iron biomarkers of the volunteers according to iron status.

	IDA (*n* = 5)	Depletion (*n* = 29)	Deficiency (*n* = 32)	Sufficiency (*n* = 38)	*P*-Value *
Women	5 (100.0)	29 (100.0)	29 (90.6)	18 (47.4)	**<0.001**
Lacto-ovo vegetarians	2 (40.0)	14 (48.3)	17 (53.1)	16 (42.1)	0.810 0.382
Vegetarianism period					
<1 year	0 (0.0)	3 (10.3)	7 (21.9)	3 (7.9)	
1–3 years	3 (60.0)	13 (44.9)	11 (34.4)	17 (44.8)	
4–10 years	1 (20.0)	9 (31.0)	5 (15.6)	13 (34.2)	
≥10 years	1 (20.0)	4 (13.8)	9 (28.1)	5 (13.1)	
Supplementation					**0.031**
Never	3 (60.0)	22 (75.9)	27 (84.5)	35 (92.2)	
1–12 times/year	2 (40.0)	7 (24.1)	1 (3.1)	1 (2.6)	
2–5 times/month	0 (0.0)	0 (0.0)	2 (6.2)	1 (2.6)	
1–6 times/week	0 (0.0)	0 (0.0)	0 (0.0)	0 (0.0)	
Daily	0 (0.0)	0 (0.0)	2 (6.2)	1 (2.6)	
Physical activity level					0.163
Low	1 (20.0)	1 (3.4)	3 (9.4)	3 (7.9)	
Medium	3 (60.0)	16 (55.2)	16 (50.0)	10 (26.3)	
High	1 (20.0)	12 (41.4)	13 (40.6)	25 (65.8)	
WHR	0.8 (0.0)	0.8 (0.0)	0.8 (0.1)	0.9 (0.1)	**<0.001**
BMI (kg/m^2^)	21.4 (3.3)	21.8 (2.9)	22.4 (3.6)	23.6 (3.6)	0.063
Body fat (%)	22.1 (7.9)	25.5 (6.3)	24.3 (7.3)	20.7 (8.8)	0.120
Body muscle (kg)	43.5 (1.9)	40.4 (2.8)	42.4 (6.7)	51.4 (10.5)	**<0.001**
Bone mass (kg)	2.3 (0.1)	2.2 (0.2)	2.3 (0.3)	2.7 (0.5)	**<0.001**
Body water (%)	57.5 (5.8)	54.8 (4.3)	55.6 (5.0)	57.9 (6.3)	0.204
Abdominal fat (%)	18.3 (8.4)	20.5 (8.1)	20.3 (7.8)	19.0 (8.8)	0.829
Abdominal muscle (kg)	24.8 (1.3)	23.3 (1.6)	24.7 (4.2)	28.5 (5.3)	**<0.001**
Leucocytes (10^3^/µL)	4.5 (1.5)	5.7 (1.0)	6.2 (1.8)	6.2 (1.8)	0.130
RBC (10^6^/µL)	4.2 (0.2)	4.5 (0.2)	4.6 (0.4)	4.7 (0.4)	**0.012**
Haemoglobin (g/dL)	10.9 (1.2)	13.5 (0.8)	14.0 (0.9)	14.6 (1.3)	**<0.001**
Haematocrit (%)	34.2 (2.4)	40.9 (2.0)	42.3 (2.7)	43.4 (3.7)	**<0.001**
MCV (fL)	82.4 (8.6)	91.3 (5.5)	92.8 (4.7)	93.1 (3.4)	**0.011**
MCH (pg)	26.3 (3.8)	30.0 (2.0)	30.6 (1.5)	31.3 (1.5)	**<0.001**
MCHC (g/dL)	31.8 (1.4)	32.9 (0.9)	33.0 (1.0)	33.8 (1.1)	**0.001**
RDW (%)	15.0 (1.6)	13.8 (1.0)	13.3 (1.4)	12.8 (0.6)	**<0.001**
Platelets (10^3^/µL)	299 (81)	243 (63)	243 (64)	224 (72)	0.244
MPV (fL)	9.8 (1.4)	10.0 (1.3)	10.1 (1.2)	10.0 (1.1)	0.972
Iron (µmol/L)	6.5 (7.2)	13.5 (7.8)	18.1 (6.4)	20.2 (8.1)	**<0.001**
Transferrin (g/L)	3.4 (0.2)	3.5 (0.6)	3.1 (0.4)	2.7 (0.5)	**<0.001**
TSAT (%)	8.0 (9.4)	15.6 (8.5)	23.8 (8.4)	29.8 (12.1)	**<0.001**
Ferritin (ng/mL)	7.1 (6.9)	9.6 (3.0)	22.0 (4.4)	65.3 (40.4)	**<0.001**
sTfR (mg/L)	1.6 (0.9)	0.8 (0.4)	0.8 (0.4)	0.7 (0.2)	**0.041**
sTfR/ferritin	389 (289)	107 (82)	39 (22)	13 (7)	**<0.001**
TBI (mg/kg)	3.4 (4.5)	7.3 (2.2)	10.7 (1.8)	14.6 (2.1)	**<0.001**

The results expressed as the mean (SD) for continuous variables and *n* (%) for categorical variables. * Kruskal-Wallis or Pearson’s χ^2^ tests. Significant differences are in bold.

**Table 4:** The gynaecological characteristics of women according to iron status.

	IDA (*n* = 5)	Depletion (*n* = 29)	Deficiency (*n* = 29)	Sufficiency (*n* = 18)	*P*-Value *
	Menstruation				
Cycle length, days	30.6 (8.2)	28.2 (2.7)	28.6 (3.4)	28.2 (2.7)	0.965
Period length, days	5.4 (0.5)	4.7 (1.4)	4.7 (1.0)	4.4 (0.5)	0.290
Heavy bleeding, days	2.8 (0.4)	2.2 (1.1)	2.0 (0.9)	1.9 (0.7)	0.128
	Hormonal contraceptives				
Contraceptive users	0 (0.0)	7 (24.1)	7 (24.1)	6 (33.3)	0.529
Contraceptive type					0.383
Oral	0 (0.0)	1 (3.4)	5 (17.2)	4 (22.2)	
Vaginal ring	0 (0.0)	5 (17.2)	1 (3.4)	2 (11.1)	
Patch	0 (0.0)	1 (3.4)	0 (0.0)	0 (0.0)	
Implant	0 (0.0)	0 (0.0)	1 (3.4)	0 (0.0)	

The results expressed as the mean (SD) for continuous variables and n (%) for hormonal contraceptive type. * Kruskal-Wallis or Pearson´s χ^2^ tests.

**Table 5:** The dietary patterns in relation to iron status.

	IDA (*n* = 5)	Depletion (*n* = 29)	Deficiency (*n* = 31)	Sufficiency (*n* = 38)	*P*-Value *
*Iron sources*					
Bread (integral)	2.0 (3.5)	2.0 (3.0)	2.0 (3.0)	2.0 (2.0)	0.838
Bread (rye)	2.0 (2.5)	1.0 (2.0)	1.0 (2.0)	1.0 (2.0)	0.578
Chickpeas	3.0 (2.0)	2.0 (0.0)	2.0 (2.0)	2.0 (1.0)	0.190
Lentils	3.0 (1.5)	2.0 (1.0)	2.0 (1.0)	2.0 (1.2)	0.168
Peas	1.0 (2.0)	1.0 (1.5)	1.0 (0.0)	1.0 (1.0)	**0.013**
Quinoa	2.0 (1.0)	1.0 (0.0)	1.0 (1.0)	1.0 (0.0)	0.403
Spinach (boiled)	1.0 (1.0)	1.0 (1.0)	1.0 (0.0)	1.0 (1.0)	0.841
White beans	1.0 (1.5)	1.0 (1.0)	1.0 (1.0)	1.0 (1.0)	0.480
*Vitamin C sources*					
Asparagus	2.0 (1.5)	1.0 (1.0)	1.0 (1.0)	1.0 (2.0)	0.634
Avocado	2.0 (1.5)	3.0 (2.0)	2.0 (2.0)	2.0 (2.0)	0.446
Blueberries	1.0 (1.5)	1.0 (1.5)	1.0 (1.0)	1.0 (2.0)	0.705
Broccoli	2.0 (2.0)	1.0 (1.0)	1.0 (1.0)	2.0 (1.0)	0.347
Cauliflower	1.0 (0.5)	1.0 (0.5)	1.0 (1.0)	1.0 (2.0)	0.511
Green beans	1.0 (1.5)	1.0 (1.0)	1.0 (1.0)	1.0 (2.0)	0.580
Kiwi	2.0 (2.0)	1.0 (2.0)	1.0 (2.0)	1.0 (2.0)	0.627
Lettuce	2.0 (3.0)	2.0 (2.0)	2.0 (2.0)	2.0 (1.0)	0.178
Melon	1.0 (1.5)	1.0 (1.0)	1.0 (1.0)	1.0 (1.0)	0.879
Orange	1.0 (3.0)	1.0 (2.0)	2.0 (0.0)	2.0 (1.0)	0.087
Orange juice	2.0 (2.5)	0.0 (2.0)	1.0 (2.0)	1.0 (1.0)	0.744
Peas	1.0 (2.0)	1.0 (1.5)	1.0 (0.0)	1.0 (1.0)	**0.013**
Potato	2.0 (0.0)	2.0 (1.0)	2.0 (1.0)	2.0 (2.0)	0.298
Pumpkin	2.0 (1.5)	1.0 (1.0)	1.0 (1.0)	1.0 (1.0)	0.503
Strawberry	2.0 (0.5)	1.0 (1.0)	2.0 (1.0)	1.0 (1.0)	0.108
Tangerine	1.0 (1.5)	1.0 (1.0)	2.0 (1.0)	1.0 (1.2)	0.679
Tomato	3.0 (2.0)	3.0 (2.0)	2.0 (1.0)	3.0 (2.0)	0.831
Zucchini	3.0 (2.5)	2.0 (1.5)	2.0 (2.0)	2.0 (1.0)	**0.005**
*Absorption inhibitors*					
Bread (integral)	2.0 (3.0)	2.0 (3.0)	2.0 (3.0)	2.0 (2.0)	0.831
Bread (rye)	2.0 (2.5)	1.0 (2.0)	1.0 (2.0)	1.0 (2.0)	0.578
Coffee	1.0 (4.0)	3.0 (4.0)	2.0 (4.0)	3.0 (4.0)	0.890
Tea	3.0 (4.0)	1.0 (2.0)	1.0 (3.0)	1.5 (3.0)	0.241
Pasta	3.0 (1.5)	2.0 (1.0)	1.0 (1.0)	1.5 (1.0)	**0.030**
Quinoa	2.0 (1.0)	1.0 (0.0)	1.0 (1.0)	1.0 (0.0)	0.415
Rice (integral)	2.0 (1.5)	1.0 (1.0)	1.0 (1.0)	1.0 (1.0)	0.754

The results expressed as the median (interquartile range). The considered categories were: 0, never; 1, 2–4 times/month; 2, 2–3 times/week; 3, 4–6 times/week; 4, once a day; 5, ≥2 times/day. * Pearson’s χ^2^ test. Significant differences are in bold.
